# Non-Invasive Optical Motion Tracking Allows Monitoring of Respiratory Dynamics in Dystrophin-Deficient Mice

**DOI:** 10.3390/cells11050918

**Published:** 2022-03-07

**Authors:** Angelika Svetlove, Jonas Albers, Swen Hülsmann, Marietta Andrea Markus, Jana Zschüntzsch, Frauke Alves, Christian Dullin

**Affiliations:** 1 Translational Molecular Imaging, Max-Planck Institute for Multidisciplinary Sciences, City Campus, 37075 Göttingen, Germany; anzhelika.svetlova@mpinat.mpg.de (A.S.); markus@mpinat.mpg.de (M.A.M.); falves@gwdg.de (F.A.); 2 X-ray Based Preclinical Imaging Technologies, Institute for Diagnostic and Interventional Radiology, University Medical Center, 37075 Göttingen, Germany; jonas.albers@embl-hamburg.de; 3 Central Breathing Control, Clinic for Anesthesiology, University Medical Center, 37075 Göttingen, Germany; shuelsm2@uni-goettingen.de; 4 Neuromuscular Disease Research, Clinic for Neurology, University Medical Center, 37075 Göttingen, Germany; j.zschuentzsch@med.uni-goettingen.de; 5 Clinic for Haematology and Medical Oncology, University Medical Center, 37075 Göttingen, Germany; 6 Multiscale Bioimaging—From Molecular Machines to Networks of Excitable Cells, Cluster of Excellence (MBExC), 37075 Göttingen, Germany; 7 Institute for Diagnostic and Interventional Radiology, University Hospital, 69120 Heidelberg, Germany

**Keywords:** Duchenne muscular dystrophy, optical tracking, breathing dynamics, *mdx* mouse model, neuromuscular disorders

## Abstract

Duchenne muscular dystrophy (DMD) is the most common x-chromosomal inherited dystrophinopathy which leads to progressive muscle weakness and a premature death due to cardiorespiratory dysfunction. The *mdx* mouse lacks functional dystrophin protein and has a comparatively human-like diaphragm phenotype. To date, diaphragm function can only be inadequately mapped in preclinical studies and a simple reliable translatable method of tracking the severity of the disease still lacks. We aimed to establish a sensitive, reliable, harmless and easy way to assess the effects of respiratory muscle weakness and subsequent irregularity in breathing pattern. Optical respiratory dynamics tracking (ORDT) was developed utilising a camera to track the movement of paper markers placed on the thoracic-abdominal region of the mouse. ORDT successfully distinguished diseased *mdx* phenotype from healthy controls by measuring significantly higher expiration constants (*k*) in mdx mice compared to wildtype (wt), which were also observed in the established X-ray based lung function (XLF). In contrast to XLF, with ORDT we were able to distinguish distinct fast and slow expiratory phases. In *mdx* mice, a larger part of the expiratory marker displacement was achieved in this initial fast phase as compared to wt mice. This phenomenon could not be observed in the XLF measurements. We further validated the simplicity and reliability of our approach by demonstrating that it can be performed using free-hand smartphone acquisition. We conclude that ORDT has a great preclinical potential to monitor DMD and other neuromuscular diseases based on changes in the breathing patterns with the future possibility to track therapy response.

## 1. Introduction

Duchenne muscular dystrophy (DMD) is the most prevalent and one of the most serious muscular dystrophies [1] for which no curative treatment is yet available [2,3]. The cause of DMD is a deletion or loss-of-function mutation in the more than two megabase large *dystrophin* gene, which leads to the loss of the muscle structure protein of the same name [4,5]. The dystrophin protein, a key component of the dystrophin-associated glycoprotein complex in skeletal muscle cells and cardiomyocytes [4], normally links the actin cytoskeleton to the sarcolemma and the extracellular matrix and thus provides mechanical stability during contraction as well as mechanosignalling to the extracellular matrix [6,7]. The absence of dystrophin leads to a contraction-induced muscle fibre damage, an altered calcium homeostasis with repeated cycles of de- and regeneration and myoinflammation until the muscle tissue is gradually replaced by fatty and fibrotic tissue [1,8,9]. Clinically, DMD patients present with progressive skeletal muscle degradation, a loss of ambulation, progressive cardiomyopathy [10] and a reduction in respiratory function [11]. Cardiopulmonary impairment is significantly involved in the premature death of patients [12,13]. The respiratory dysfunction results from morphological changes of the diaphragm and the auxiliary respiratory muscles [14] and thereby must be routinely assessed in DMD patients to determine the optimal time point for pulmonary respiratory aids and support. Although the standard treatment with glucocorticosteroids and non-pharmacological approaches including an improved management of cardiac function and respiratory support have increased survival of DMD patients by up to 10 to 20 years, the disease is still fatal between the third and fourth decades of life [15,16] Therefore, considerable efforts are undertaken towards targeted mitigation of the causative genetic mutation or compensating for the lack of dystrophin such as read-through [17], exon-skipping [18], vector-mediated gene therapy [19], CRISPR/Cas9 gene editing [20] and cell therapy [21], all aiming to decrease morbidity and mortality and increase the life quality of these patients [22]. 

The most common mouse model for DMD is the well-established *mdx* (C57 BL/10 ScSn-Dmdmdx/J (Bl10/*mdx*))-mouse [23]. The *mdx* (X-chromosome-linked muscular dystrophy) mouse was discovered spontaneously in a C57 BL/10 ScSn colony [24] and is characterised by a nonsense mutation in Exon 23 which results in a premature stop codon and absence of dystrophin protein [4,25,26,27]. Even though *mdx* animals have a milder phenotype compared to DMD patients, they exhibit a characteristic DMD pathology of the diaphragm early in the disease phase [24]. The myodystrophic processes of the *mdx* mouse increase over its lifetime [28].

For optimised use of *mdx* animals, sensitive and standardised preclinical measurement methods have been developed by the TREAT-NMD network (Translational Research in Europe for the Assessment and Treatment of Neuromuscular Disease; http://www.treat-nmd.eu, accessed 30 December 2021). Terminal or paraclinical measurement parameters such as serum creatine kinase (CK), histological changes in the skeletal muscle and the expression of mediators at the mRNA level are recommended. Other preclinical assessments in *mdx* mice include motor tests such as determination of grip strength and running wheel analyses. The recommended procedures for respiratory system evaluation are spirometric measurements with a pneumotachometer, intratracheal end-point ventilation and whole-body plethysmography [29].

Respiratory muscle weakness is a known consequence of many neuromuscular diseases. Patients with conditions such as amyotrophic lateral sclerosis (ALS), certain myopathies, myasthenia gravis and muscular dystrophies, including DMD, are found to have abnormal breathing patterns and changes in respiratory dynamics [30,31,32,33]. Latter observation makes monitoring DMD disease progression based on the respiratory dynamics desirable for both, humans and mouse models and has motivated this study.

One option to monitor respiratory dynamics is whole-body plethysmography, which previously has been used to analyse DMD mouse models [34,35,36,37,38,39]. An alternative method is X-ray based lung function (XLF) which relies on planar low dose X-ray imaging [40,41,42]. While the method to this day has been used primarily to monitor mouse models of chronic and inflammatory pulmonary diseases, it, nevertheless, has been shown to be suitable for tracking the progression of respiratory function [41]. However, XLF relies on the use of ionising radiation. While the received dose is relatively small (6 mGy), due to accumulation, XLF is not suitable for long-lasting follow-up studies with repeated measurements. Therefore, the aim of the present study was to establish an alternative optical method that is able to recognise changes of the respiratory dynamics of the *mdx* phenotype. To achieve this, we developed the optical respiratory dynamics tracking (ORDT) based on camera-enabled tracing of chest surface markers and compared the results with XLF measurements in *mdx* and wt mice.

ORDT was found to have the following advantages: (1) easy to perform, (2) non-invasive, (3) low cost and (4) featuring commonly available tools and equipment. Moreover, ORDT was successfully able to reproduce XLF derived data, showing a faster expiration in *mdx* mice compared to wt controls. Additionally, ORDT depicted a clear two-phase expiration pattern present in both mdx and wt mice, and revealed distinct differences in the two-phase expiration behaviours, which was not found in the XLF measurements.

## 2. Materials and Methods

### 2.1. Mouse Model and Experimental Design

Mdx mice (C57 BL/10 ScSn mdx) used for breeding were kindly provided by Ralf Herrmann (University of Essen, Germany). Equal numbers of male and female mice were used in mdx and healthy [C57 BL/10 ScSn] control groups. The longitudinal experiment spanned 12 weeks, the measurements were performed on mice at 5, 7, 9, 11, 13 and 17 weeks of age. For the initial measurement at five weeks 5 animals in each group were recruited (*n* = 5) and 1 wt, 1 mdx mice were sacrificed directly after the measurement for model validation by histological assessment (Figure S1). At 7 weeks, 3 additional mice were added to each group for all following measurements (*n* = 7). Upon the completion of the timeline, all of the mice continued in another experiment unrelated to this study. The summary of the body masses can be found in Figure S2. For XLF and ORDT measurements, the animals were anaesthetised with 2–3% isoflurane, airflow of 1 L/min of 50:50 mix of air and oxygen. The anaesthesia was titrated to maintain the respiratory intervals at approximately 1400 ms. This interval was chosen due to limiting acquisition frame rate (30 Hz) of the X-ray detector used in the XLF analysis. An additional set of ORDT measurements were performed at approximately 800 ms breathing intervals to compare the effect of deepness of anaesthesia on the functional parameters. ORDT and XLF readings were performed sequentially on the same day on the same animals in both groups. First ORDT measurement was performed on the mouse in supine position, the markers were then removed, and the CT scans were then performed without repositioning the mouse. The experiments were approved by the responsible ethics committee of the state of Lower Saxony, Germany and performed in accordance with the ethical guidelines of the national animal protection law. The colony was maintained by crossing *mdx* males with *mdx* females and bred at the central animal facility of the University Medical Center Göttingen. All mice were housed in plastic individually ventilated cages and maintained on a regular diurnal lighting cycle (12:12 light:dark) with ad libitum access to standard rodent food and water.

### 2.2. Optical Respiratory Dynamics Tracking (ORDT)

For the standard ORDT the breathing motion of anaesthetised mice was recorded by a camera (Basler acA640-90 gc, Basler AG, Ahrensburg, Germany; Pentax Cosmicar TV Lens 25 mm 1:1.4, Ricoh, Tokyo, Japan) acquiring images with 600 by 400 pixels for over 10 s at 100 frames per second (fps) in 8-bit grey value format. Paper markers with a printed black cross-hair pattern (3 mm side length and 1 mm centre diameter) were used. They were placed on the thoracic-abdominal region of the animal and attached to the fur of the mice with double-sided tape. The markers in the video feed were located by a Laplacian of Gaussian particle detection and tracked by Linear Assignment Problem algorithms implemented in the TrackMate plugin for ImageJ [43]. Calculation of the expiration constant was performed in the latest version of XLF software [40].

For smartphone-based ORDT (iORDT), an iPhone 12 (Apple Inc., Cupertino, CA, USA) was used. The breathing motion was recorded freehand in HD 1920 by 1080 pixels using 60 and 240 fps modes. The resulting frame rate for the 240 fps was approximately 177 fps as stated by the smartphone device. The acquired video was motion-corrected (see Supplement for further details) the resulting image sequence was then entered to the same analysis pipeline as in the ORDT approach.

### 2.3. X-ray Lung Function Measurement

Low dose planar cinematic X-ray images were acquired in anesthetised mice using an in vivo microCT (QuantumFX, Perkin Elmer, Waltham, MA, USA) using the following parameters: field of view 20 mm × 20 mm, tube voltage 90 kV and tube current 40 µA. 1024 images were recorded continuously at 30 fps. Mice were scanned unrestrained and as with the ORDT set up. The X-ray transmission over time in the lung area was measured and the expiration constant was calculated using latest version of the XLF software [40].

### 2.4. Statistical Analysis

All calculated parameters were analysed by mixed-effects model with *p* < 0.05 significance level. Šídák’s multiple comparisons test was performed to find the differences between mdx and wt groups on individual measurement days. Statistical analysis was performed in Graphpad Prism version 9.2.0 (GraphPad Inc., San Diego, CA, USA).

## 3. Results

### 3.1. Optical Respiratory Dynamics Tracking (ORDT) Workflow and Its Comparison to XLF

**Figure 1:**
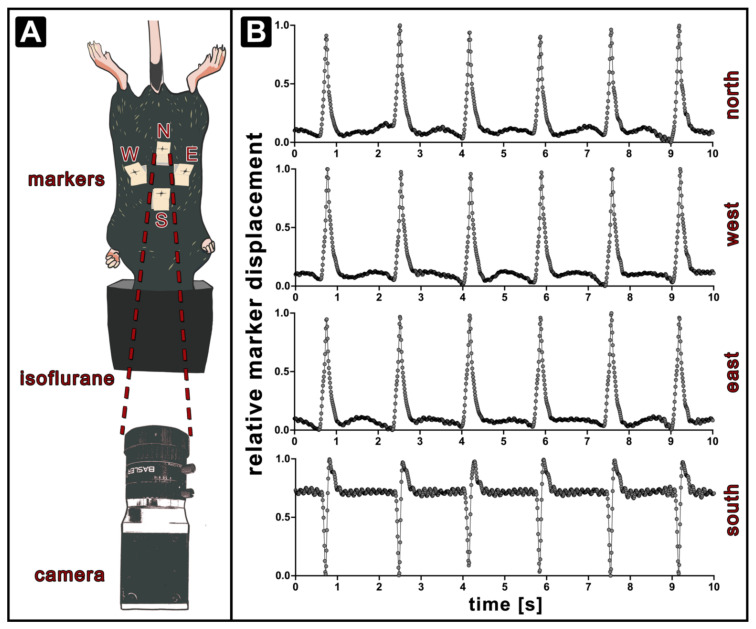
Setup for optical respiratory dynamics tracking (ORDT). The mouse is placed on a plastic rail with the chest and abdomen facing upwards and the nose exposed to isoflurane gas through a face mask. Four markers are positioned on the fur of the mouse and their movement is recorded by a camera positioned 25 cm away at an angle of 40°. The paper markers are placed on skin/fur at different sites. South marker (S) is trapped on the skin above the sternum. East (E) and west markers (W) are both placed at the location of the 12th rib and north marker (N) is positioned on the area of the abdomen (**A**). Exemplary marker traces of a wt mouse at 38 days from birth after the pre-processing procedure show that the modulations in N, E and W markers are following the same shape, while the S marker traces a different motion (**B**).

To perform the optical measurement, the following set-up was established (Figure 1A). The mouse was positioned lying on its back with its nose exposed to isoflurane anaesthesia via a mask. The camera was placed 25 cm cranial and 10 cm anterior to the anaesthetised mice and at 40° facing the abdomen. Four paper markers were positioned in the thoracic-abdominal region of the mouse in order to capture possible phase shifts in the chest motion across different quadrants of the recorded area. The markers were designed with a visible cross-hair pattern for easy camera detection. Thus, four markers were attached: abdominal marker placed on the umbilicus (north, N), sternum marker (south, S) and the 12th rib region on each side (east, E, west, W). In order to parametrise the optical recordings, the markers had to be identified and traced over time. For every marker ∆y and ∆x were calculated as anterior-posterior and sinister-dexter displacements over time. It was further assumed that the total displacement of the markers in each location represents the state of the chest expansion. Due to the shape of the mouse and the marker placement, the analysis cannot be performed considering the displacement in the original *y* or *x* axis alone. Therefore, the data for each marker individually was projected into the cardinal direction of the movement (*y*’) using a Karhunen–Loéve Transformation. To compensate for low-frequency artefacts associated with setup vibration, the resulting traces were filtered, corrected for background and normalised. We applied an adaptive moving average filter with a filter window of *w*. Since a standard moving average filter ***F_m_*** may alter the shape of the curve, a weighted moving average filter ***F_w_*** was applied to penalise points that represent the breathing peak. This approach successfully removed background variations without interfering with the shape of the peak.

(1) $$F_{m}\left(t\right)\;=\;\frac{1}{w}\;\overset{w/2}{\underset{i=-w/2}{\sum }}y^{\prime }\left(t+i\right)$$

(2) $$F_{w}\left(t\right)\;=\;\frac{1}{G\left(t\right)}\;\sum_{i=-w/2}^{w/2}\left(\frac{y^{\prime }\left(t+i\right)}{\left[\left|y^{\prime }\left(t+i\right)\;-\;F_{m}\left(t+i\right)\right|\;+\;0.1\right]}\right)$$

(3) $$G\left(t\right)\;=\;\sum_{i=-w/2}^{w/2}\left(\left|y^{\prime }\left(t+i\right)\;-\;F_{m}\left(t+i\right)\right|\;+\;0.1\right)$$

***F_w_*** was then subtracted from y’ and the result was scaled to a dynamic range between 0 and 1. Breathing peaks were identified using a threshold of 30%. The starting point and the end-point of a breathing event were defined as local minima in the approximated 2nd derivative of the function prior to the breathing peak. Examples of the resulting exemplary relative marker displacement (rmd) traces of a wt mouse at 5 weeks (38 days) of age from each marker are shown in Figure 1B. The appearance of the breathing peaks in N, W and E traces is consistent, confirming that the cardinal displacement obtained by the performed procedure represents the expanding breathing motion. Since the S marker is located more cranial than the rest of the markers but is still at the caudal end of the sternum, its movement is affected by the movement of the thorax and the abdomen. The biphasic behaviour can be attributed to the placement of the marker on the chest region where the diaphragm passes twice in a single breathing cycle. Additionally, a more prominent pulse wave generated by the heart is seen as a high-frequency modulation in the baseline.

**Figure 2:**
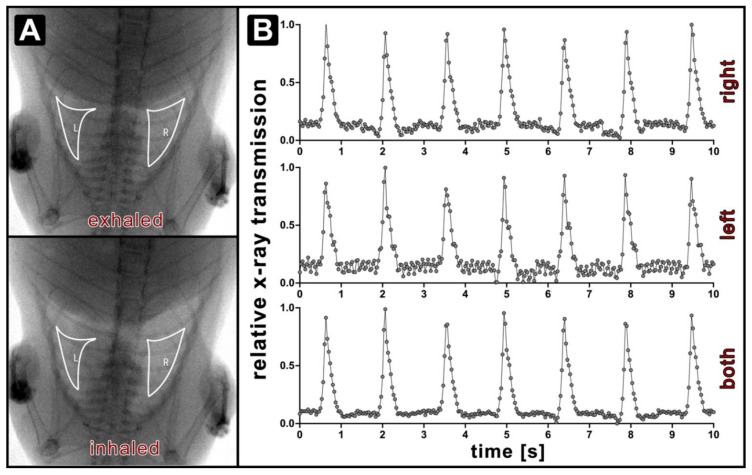
Exemplary XLF measurement of a wt mouse at 38 days from birth displaying representative images of exhaled and inhaled states as seen in the planar X-ray projection video with right (R) and left lung (L) regions defined (**A**) and the corresponding calculated relative X-ray transmission traces obtained from right, left, and both lungs averaged (**B**).

The breathing traces obtained by ORDT were compared to XLF breathing measurements, which are based on the modulation of the averaged X-ray transmission in the lung region. An example of XLF recording from a wt mouse is shown in Figure 2 with selected X-ray transmission regions of interest in right and left lungs displayed in panel A. Obtained average transmission traces are shown in panel B. It is evident that the shape of the breathing events in the traces obtained by ORDT is closely recapitulated by the XLF derived traces. High-frequency modulation present in the baseline of the S trace can be spotted in the XLF trace derived from the left lung region as well due to heart proximity.

### 3.2. Expiratory Dynamics Measured by ORDT Reproduce Expiratory Constant Differences Found by XLF

**Figure 3:**
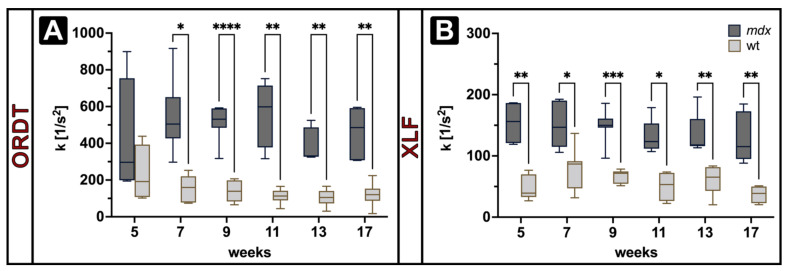
Expiration constant (*k*) derived from ORDT (**A**) and XLF (**B**) measurements in *mdx* and wt mice (*n* = 5 at 5 weeks, *n* = 7 onwards) at 1400 ms breathing intervals. ORDT shows a significantly higher *k* in *mdx* group compared to wt at all observed time points except week 5. Similarly, XLF derived *k* is significantly higher in *mdx* group compared to wt at all observed time points. Both XLF and ORDT did not show a significant difference in *k* across weeks in each group. Box annotations represent the median and 5–95% intervals. Statistical significance between groups on each measurement determined by Šídák’s multiple comparisons test * *p* < 0.05, ** *p* < 0.01, *** *p* < 0.001, **** *p* < 0.0001.

To validate whether the ORDT approach is able to detect the DMD/*mdx* typical phenotype of respiratory neuromuscular weakness and to establish its sensitivity towards respiratory dynamic changes, ORDT derived traces were compared to an established XLF measurement [40]. According to the existing XLF procedure, all expiration segments from a single measurement were combined and a function (Equation (4)) was fitted ensuring the coefficient of determination to be >0.9. Expiration constant (*k*) was calculated for the XLF derived expiration segments in the right lung and for ORDT derived expiration segments from N, W and E markers. There were no significant differences between N, W, E *k* values (data not shown), therefore only the N marker is further reported on. Figure 3 shows the computed values for XLF (right lung) and ORDT (N) in *mdx* and wt mice over the period of 5 to 17 weeks of age.

(4) $$A\left(t\right)\;=\;A_{0}e^{-k\cdot t^{2}}+b$$

XLF derived *k* was found to be significantly higher in *mdx* mice compared to wt at all observed timepoints, with higher *k* indicating steeper expiratory function (Figure 3B). ORDT derived *k* was found to be significantly higher in *mdx* mice compared to wt from 7 to 17 weeks of age (Figure 3A). For both XLF and ORDT no significant changes with time were observed in either *mdx* or wt groups. 

### 3.3. ORDT Identifies Two-Step Expiratory Behaviour which Differs between mdx and wt

**Figure 4:**
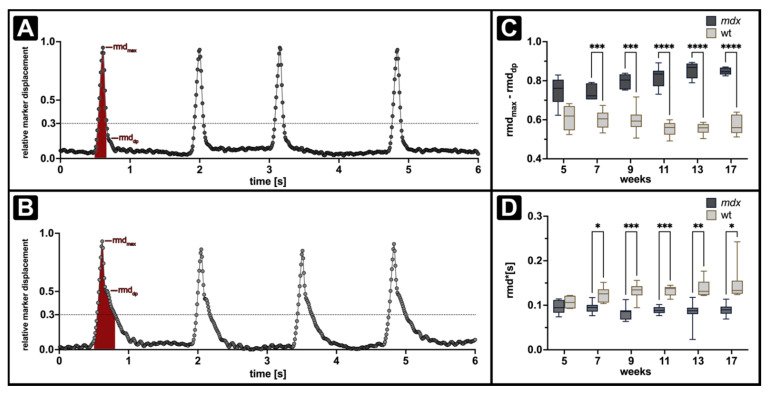
Longitudinal measurements of fast expiratory phase and area under the curve in *mdx* and wt mice at 1400 ms breathing intervals. A, B show representative *mdx* (**A**) and wt (**B**) north marker ORDT derived relative displacement traces, as well as definitions of the computed AuC (filled red area) and relative fast expiratory phase amplitude (rmd_max_-rmd_dp_). Relative amplitude of the fast expiratory phase (**C**) is significantly larger in the *mdx* group compared to wt, in line with this AuC (rmd·(s)) (**D**) is significantly larger in wt compared to *mdx*. Box annotations represent the median and 5–95% intervals. Statistical significance between groups determined by Šídák’s multiple comparisons test * *p* < 0.05, ** *p* < 0.01, *** *p* < 0.001, **** *p* < 0.0001.

The simple calculation of the expiration constant revealed the differences in the expiratory dynamics between *mdx* and wt in both XLF and ORDT measurements. However, when comparing the shapes of the breathing peaks obtained by ORDT (Figure 1B) and XLF (Figure 2B) it is evident that ORDT derived expiratory segments display a two-step behaviour with a fast descending phase immediately at the beginning of expiration followed by a slow phase. The same phenomenon cannot be observed by XLF due to the limitation of the sampling rate which is 3 times smaller than that of ORDT. Figure 4 shows representative traces of *mdx* (A) and wt (B) mice obtained by ORDT. Evidently, the two-phase expiratory behaviour is observed in both phenotypes, however, the distribution of the phases differs with *mdx* slow expiratory phase beginning a lot later in the breathing event which we termed the ‘dampening point’. Since the function native to the XLF analysis pipeline assumes a single Gaussian (Equation (4)), it may not be the best representation of the two-phase expiration data obtained by ORDT. Therefore, the dampening point was defined as the relative marker displacement (rmd) corresponding to the absolute minimum in the third derivative of a fitted sum of Gaussians (Equation (5)). Furthermore, the relative amplitude of the fast expiratory phase was quantified as the difference between maximum relative marker displacement (rmd_max_) and the relative marker displacement of the dampening point (rmd_dp_) (Figure S3). In line with the expiration constant observation, amplitude of the fast expiratory phase was found to be significantly larger in *mdx* mice when compared to wt at all observed time points, except for week 5 (Figure 4C).

(5) $$D\left(t\right)\;=\;A_{0}e^{-k1\cdot t^{2}}+L_{0}e^{-k2\cdot t^{2}}+b$$

Additionally, the area under the curve (AuC) was calculated by integration of the aforementioned function from the last minimum before the breathing event to 30% of the maximum at the end of the breathing event. AuC was found to be significantly higher in wt mice compared to *mdx* mice at all observed time points except at 5 weeks (Figure 4D).

The symmetry of the breathing in *mdx* mice is of particular interest since DMD affected patients are known to exhibit signs of paradoxical breathing [44,45,46], which is defined by the changes in phase between the chest and the abdomen. Thus, simultaneity of the local maxima of the inspiration was analysed by means of ORDT. Here particularly both the left-right symmetry across the sagittal plane of west and east markers and the cranial-caudal continuity of the south and north markers were of interest. No significant phase shifts between any of the marker positions in either right-left or cranial-caudal movement for either of the groups were observed. With these findings, no evidence of paradoxical or otherwise notable breathing asymmetry was detected at all observed time points (data not shown). Additionally, we found no significant differences in the amplitude of displacement between *mdx* and wt for any of the markers or the calculated inspiration times (data not shown). When comparing different breathing intervals, there were no significant differences between 1400 ms and 800 ms breathing intervals in the same group in any of the measured parameters (Figure S4).

### 3.4. iORDT Reveals the Importance of the Sampling Rate in Diseased Phenotype Differentiation

**Figure 5:**
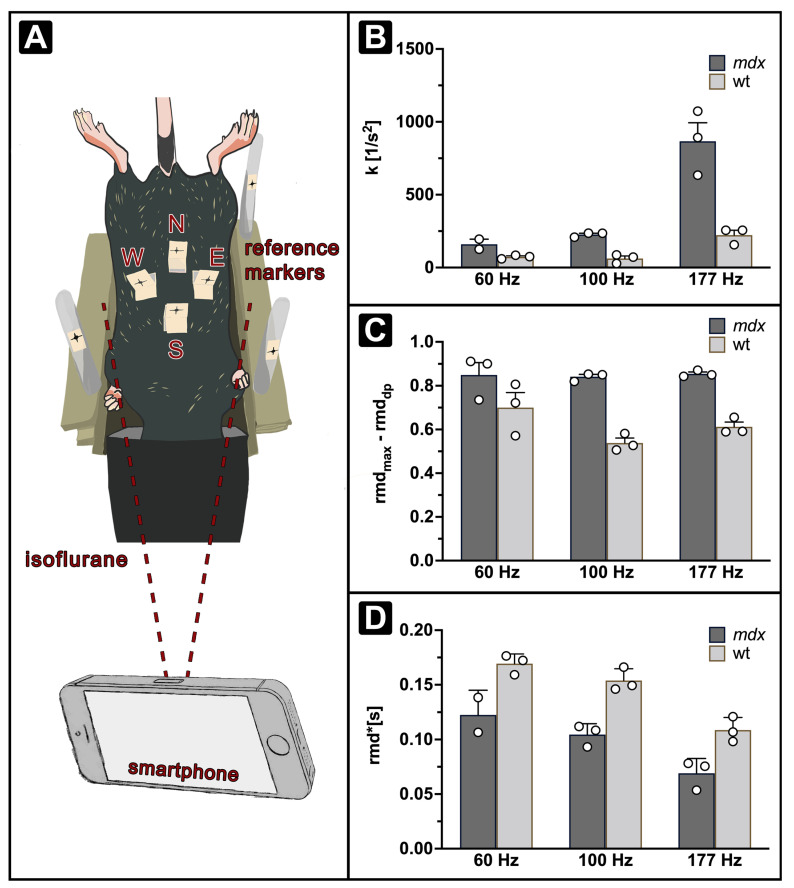
Comparison of parameters derived across different recording frequencies with ORDT (100 Hz) and iORDT (60, l77 Hz) at 1400 ms breathing intervals. Visual representation of iORDT setup using 3 additional reference markers positioned on poles around the mouse holder (**A**). *k* (**B**) and AuC (**D**) change significantly across different recording frequencies while fast expiratory phase-amplitude (**C**) remains stable.

The possibility to simplify the ORDT approach further was explored, and a smartphone-compatible version of the protocol was developed (iORDT). A smartphone was used to provide the following advantages: (1) no additional equipment is required, (2) the recordings are streamed directly to the device’s memory and (3) it has a capability of recording with high sampling rates. The setup was modified by including stationary markers for the possibility of motion correction (Figure 5A). The parameters expiration constant *k* (Figure 5B), relative fast expiratory phase amplitude (Figure 5C) and AuC (Figure 5D) were derived as described above from 60 and 177 Hz smartphone recordings. *k* has shown better segregation of the phenotypes at 177 Hz, while at 60 Hz this difference was no longer evident. Additionally, the changes in *k* across different recording frequencies were prominent. Amplitude of the fast expiratory phase remained segregated between phenotypes at all recorded frame rates. AuC has remained different between *mdx* and wt mice, similarly to *k* it changed across different recording frequencies.

## 4. Discussion

Here we developed a novel, simple, low cost and non-invasive approach to track respiratory dynamics based on optical imaging utilising a camera and markers placed on the thoracic-abdominal region in the *mdx* mouse model of Duchenne muscular dystrophy. ORDT was for the first time applied to successfully differentiate breathing behaviour of diseased *mdx* mice from healthy controls. When ORDT was compared with the established XLF method and analysed according to the same principle [40,41,42], we found significantly steeper expiration in *mdx* mice compared to controls at all measured time points beginning with the 7th week from birth as observed by the calculated expiration constant (*k*). Therefore, ORDT was able to reproduce XLF results without the need for X-ray dose exposure. Evidently, while XLF relies on changes in X-ray attenuation inside the lung, essentially quantifying the shifts in lung transparency (how much air is in the lungs) during breathing, ORDT describes the dynamic expansion state of the chest and abdomen. Fibrotic remodelling of the diaphragm with loss of function is a known consequence of *mdx* disease [24,47,48,49]. Additionally, previous findings report on anatomical changes of the skeletal structure such as increased spine curvature (kyphosis) [50]. Others further report remodelling in *mdx* intercostal muscles and recruitment of accessory musculature for breathing assistance with the progression of the disease [51,52]. Therefore, a steeper expiratory phase in *mdx* mice displayed by ORDT is likely an observed cumulative effect due to pathological remodelling on the microscopic level and changes in macroscopic anatomy leading to altered pressure differentials and musculature contributions, which may not be captured by XLF to the full extent.

Interestingly, ORDT has shown a distinct two-phase expiration pattern in both *mdx* and wt mice which was not evident in XLF measurements. While the wt mice had almost equally distributed fast- and slow-phases, *mdx* mice displayed a less prominent slow-phase and a significantly larger fast-expiration-phase as shown by the dampening point calculation. Previously, reports on post-inspiratory contractile electrical activity of the diaphragm have been made [53,54,55,56]. Pellegrini et al. state that this activity is responsible for the acute dampening of the elastic recoil produced by the lung during expiration and actively prevents its collapse [53]. It is feasible that with the weakening of the diaphragm muscle in *mdx* mice, while the inspiratory motion remains intact, potentially due to use of accessory inspiratory muscles, the post-inspiratory contractility of the diaphragm appears critically affected and is overwhelmed by the elastic recoil of the lung.

The data from functional breathing measurement in *mdx* mice is not equivocal. Several sources describe a reduction in tidal volumes in *mdx* at 2, 3 and 6 months of age [36,37,39]. Quindry et al. report no functional differences between 2-month-old *mdx* and wt mice [39]. Roberts et al. have shown no difference in inspiration time between *mdx* and wt mice at 4, 9 and 16 weeks of age, but found that the peak expiratory flow was significantly higher in *mdx* with retained expiration times at the observed time points [57]. Other methods to monitor breathing dynamics include respiratory inductance plethysmography (RIP), which uses elastic resistance bands placed around the top chest and the abdomen [58,59,60]. The method was recently employed in the golden retriever dystrophy model [61]. The expiratory flow ratios of young and adult dogs were found to be elevated, pointing to increased expiratory recoil. The faster termination of the respiratory cycle detected with ORDT with expiration constant measurement is in line with these observations.

It should be noted that the periods of inspiration and expiration in the ORDT approach are defined by the maximum and minimum positions of the relative marker displacement. It is known that the breathing contractile musculature generates pressure gradients that lead to flow of air in and out of the lungs. Thus, inspiration/expiration phases defined for ORDT are not strictly the same phases defined by classical volume and flow representations. Therefore, a further investigation with plethysmography calibration is necessary to elucidate how the different expiratory behaviour between *mdx* and wt seen in ORDT is represented in a functional sense.

In this study, all ORDT derived measurements were not significantly different on the first measurement day at 5 weeks of age since large variability in the values was detected. It is known that *mdx* diaphragms experience peak inflammation at 4 weeks for age [62], it is therefore feasible that the effect on the diaphragm immediately following inflammation was not fully established in all animals by week 5 or a permanent fibrosis was present. The amplitude of the fast expiratory phase in the *mdx* mice appears to follow an upward trend over time. While no progression in the observed time frame has been reported previously, a further investigation over a longer time period may be helpful in elucidating the role of the two-phase expiration on overall course of the disease.

Since many neuromuscular disorders such as amyotrophic lateral sclerosis (ALS), certain myopathies and myasthenia gravis also display abnormal breathing dynamics [30], we believe that a comparative study using ORDT in different mouse models of neuromuscular disorders would be of great interest. Additionally, the approach may prove to be feasible in human subjects. Great care has to be taken when addressing translatability into patients, since the relationship between chest/abdomen surface and lung pressure-volume dynamics largely vary from a highly compliant mouse chest [63]. Further, we have shown that ORDT is possible with a smartphone. The use of personal devices such as smartphones and tablets has been a rising interest in research and medicine. The opportunity to diagnose and monitor certain diseases directly from home by a commonly available personal device is of large interest.

To conclude, we established a non-invasive and easy to use method, ORDT, that is able to distinguish diseased *mdx* phenotype by breathing dynamics. We were able to derive two distinct expiration phases that differ between *mdx* and wt mice. Further investigation will be made in order to understand how these observations correspond to functional lung volume and airflow. Since the approach is based on the assessment of the dynamics of the body surface, thereby detecting abnormal breathing patterns observed in many other neuromuscular disorders, ORDT has future potential to become an easy technique for preclinical monitoring of disease severity as well as tracking the response and efficacy to therapeutic intervention.

## Data Availability

The data that support the findings of this study are available from the corresponding author upon reasonable request.

## Supplementary Materials

The following supporting information can be downloaded at: https://www.mdpi.com/2073-4409/11/5/918/s1, Figure S1: Validation of the mdx model by histological and CT assessments. Figure S2: Summary of mouse body mass. Figure S3: Detailed representation of the fast expiratory phase amplitude calculation. Figure S4: Comparison of expiration constant k (top) and relative fast expiratory phase amplitude (dp) (bottom) at 800 ms and 1400 ms breathing intervals.
