# Supplementary material for: Non-Invasive Optical Motion Tracking Allows Monitoring of Respiratory Dynamics in Dystrophin-Deficient Mice
Source: Cells. 2022 Mar 7;11(5):918. doi: 10.3390/cells11050918 (PMC8909479; doi:10.3390/cells11050918)
Supplement: Supplementary file 1 [file cells-11-00918-s001.zip › cells-1559960-supplementary.pdf]

cells

MDPI

Supplement:

Smartphone video motion correction:

Three additional stationary markers were placed on the animal stage and were used for correcting unwanted motion associated with the recording. The recorded movies were converted into a stream of 8 bit grayscale *.ppm images using FFmpeg (ffmpeg.org). A custom-made software was used for jitter and background motion correction. To this end, a region of interest was interactively placed at a static part of the scene. The drift of this region in the following frame was calculated exploiting the Fourier shift theorem. The correlation of the two regions was calculated in Fourier space and the drift was defined as the deviation of the peak value of the correlation from the centre of origin. To further improve the precision of the approach, the local area of the correlation peak was up-sampled by a factor of 10 allowing to estimate the drift vector at a subpixel level. Prior to calculating the drift of frame N with respect to frame 0, previously calculated drift of frame N-1 was applied. With this approach even large total drifts could be evaluated without enlarging the search area and the reference region kept constant, both facilitating the speed of the algorithm.

Histology and retrospective gated CT:

The mice were sacrificed using a CO_2 overdose in combination with a cervical dislocation. The lung and the diaphragm of each mouse were explanted, fixed in 4% paraformaldehyde, and embedded in paraffin for further histological analysis. 2 µm thick paraffin sections of lung and diaphragm were used for staining procedures and subsequent imaging by light microscopy with an Axiovert 200 microscope (Zeiss). Hematoxylin and eosin (H&E) staining was performed according to the manufacturer's protocol (Merck). A Masson's trichrome staining kit (Sigma Aldrich) was used to stain for collagenous connective tissue fibres. Retrospectively gated lung CT scans were acquired with: field of view 20 × 20 mm^2, tube voltage 90 kV, tube current 100 µA, total rotation angle of 720 degrees and 1020 projections. Projections were sorted according to chest movement to suppress motion artefacts. The volume datasets predominantly representing the expiration phase with a matrix of 512 x 512 x 512 pixels were reconstructed. These data sets were then rendered and the lung envelope was segmented with histogram thresholding using custom 3D rendering software (Scry v7.0, xPIT Goettingen)

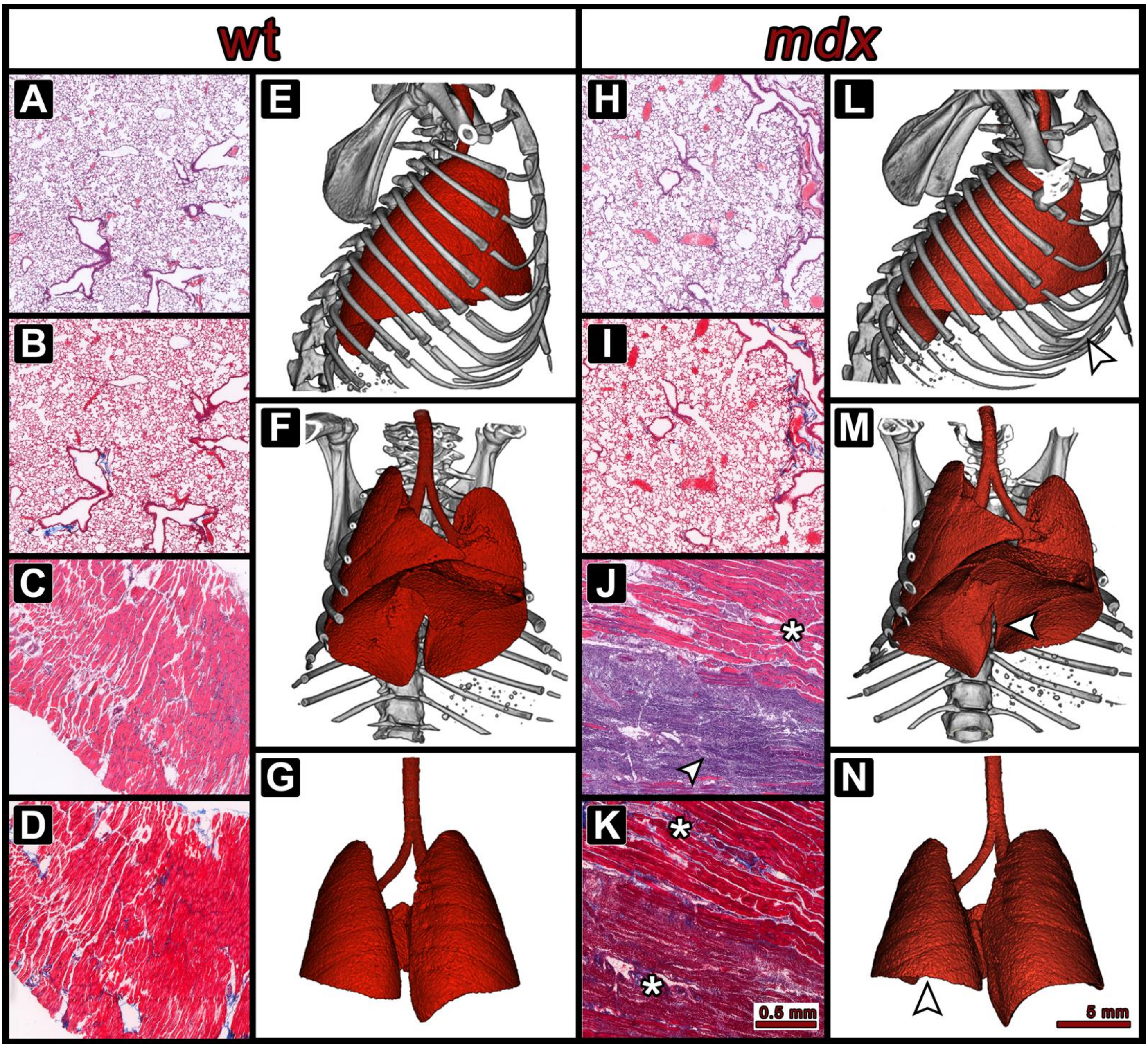

Figure S1. Validation of the mdx model by histological and CT assessments. Histological assessment and CT reveal diaphragm remodelling and bone deformation but no lung pathology in mdx mice. Histochemical staining of lung (**H&E**: **A**, **H**; MTS: **B**, **I**) and diaphragm (**H&E**: **C**, **J**; MTS: **D**, **K**) paraffin sections obtained from wt (left) and mdx (right) mice at 38 days of age. No signs of fibrotic remodelling or immune cell infiltration are found in the lungs of mdx and control mice. Diaphragms of the mdx mice exhibit signs of inflammation and tissue necrosis as characterised by invading immune cells (J, arrowhead) as well as degenerative tissue remodelling with transverse striations (J, asterisk *) and high collagen content (K, asterisk *) shown in blue and muscle fibres shown in bright red. Representative renderings of retrospectively gated chest CTs in exhaled state of a wt and mdx mouse at 38 days of age displaying different parts of the thorax : lung and rib cage from the right (E, **L**), lung and partial rib cage from the front (**F**, **M**), lung alone from the back (**G**, **N**). The mdx mice exhibit alterations of the bone structure of the chest: ballooning of the thorax with a sternal protrusion (L, arrow) as well as an increased front-to-back curvature of the upper spine identified as the beginning of kyphosis. Abnormal elevation of the left hemidiaphragm (**N**, arrow) in comparison to control (**G**) as well as a rise in the central diaphragm tendon are seen in the mdx (**M**).

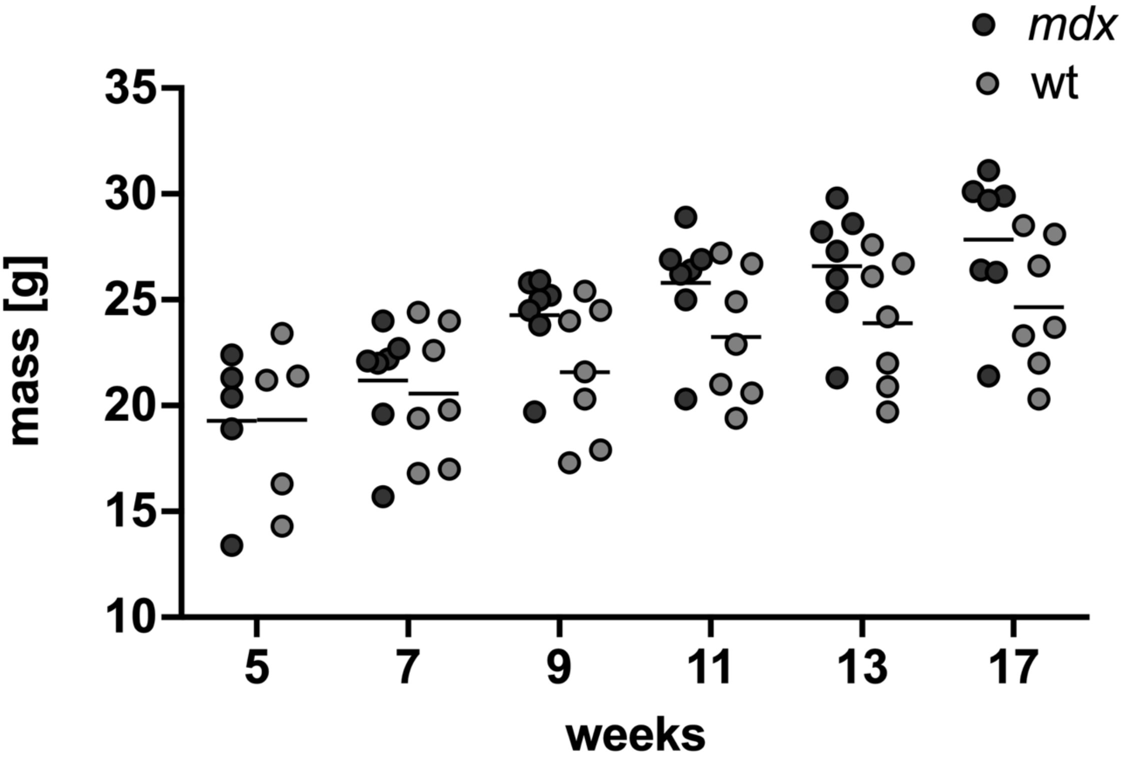

Figure S2. Summary of mouse body mass. Mixed effect analysis showed that there's significant difference (p<0.0001) in body masses between mdx and wt across reported time.

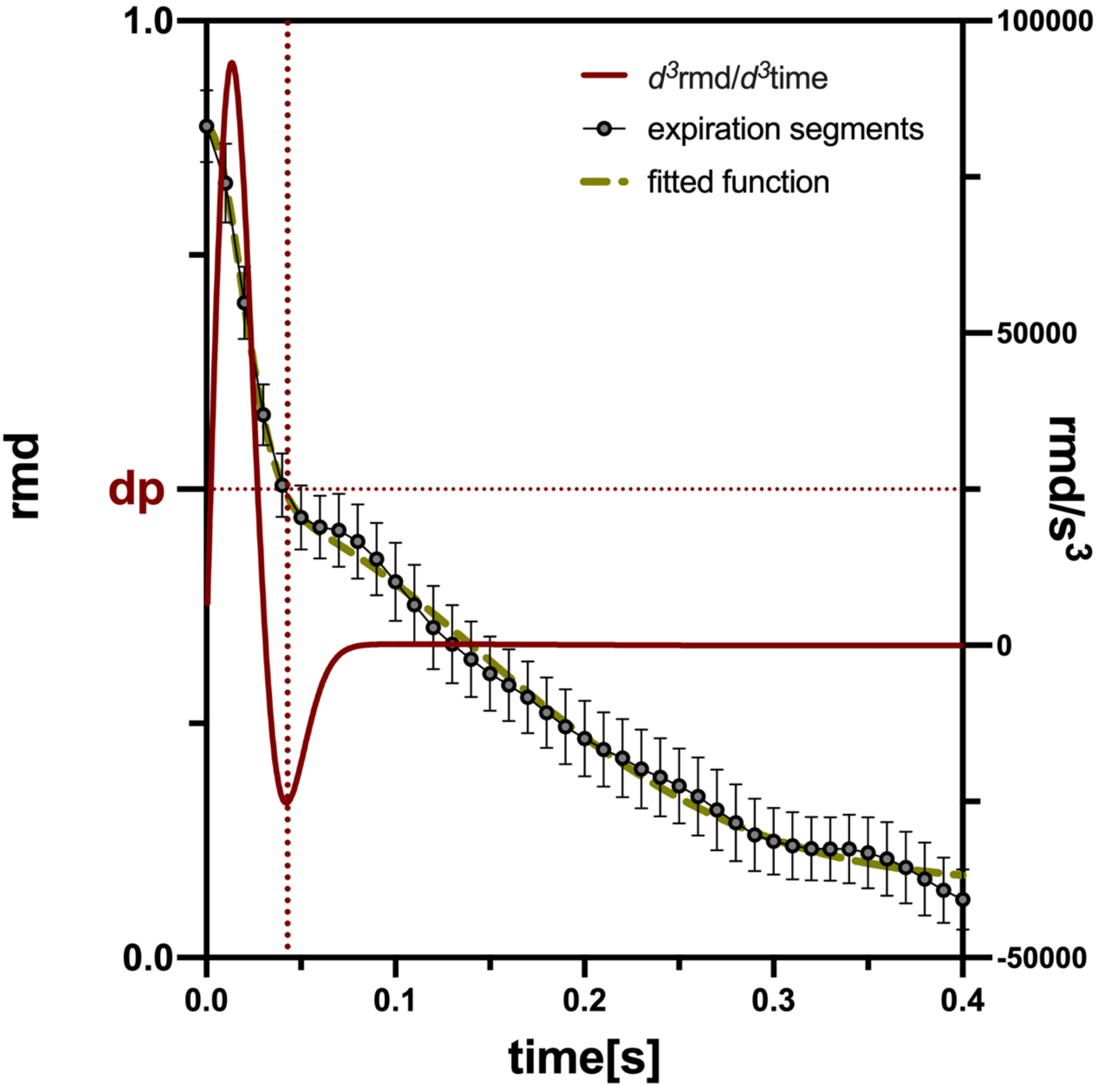

Figure S3. Detailed representation of the fast expiratory phase amplitude calculation. The third derivative (red solid line) was calculated for the sum of two Gaussians function (green dashed line) fitted to the expiration segments (grey points). Dotted red lines represent the dampening point (dp) on the original curve defined as the minimum in the third derivative.

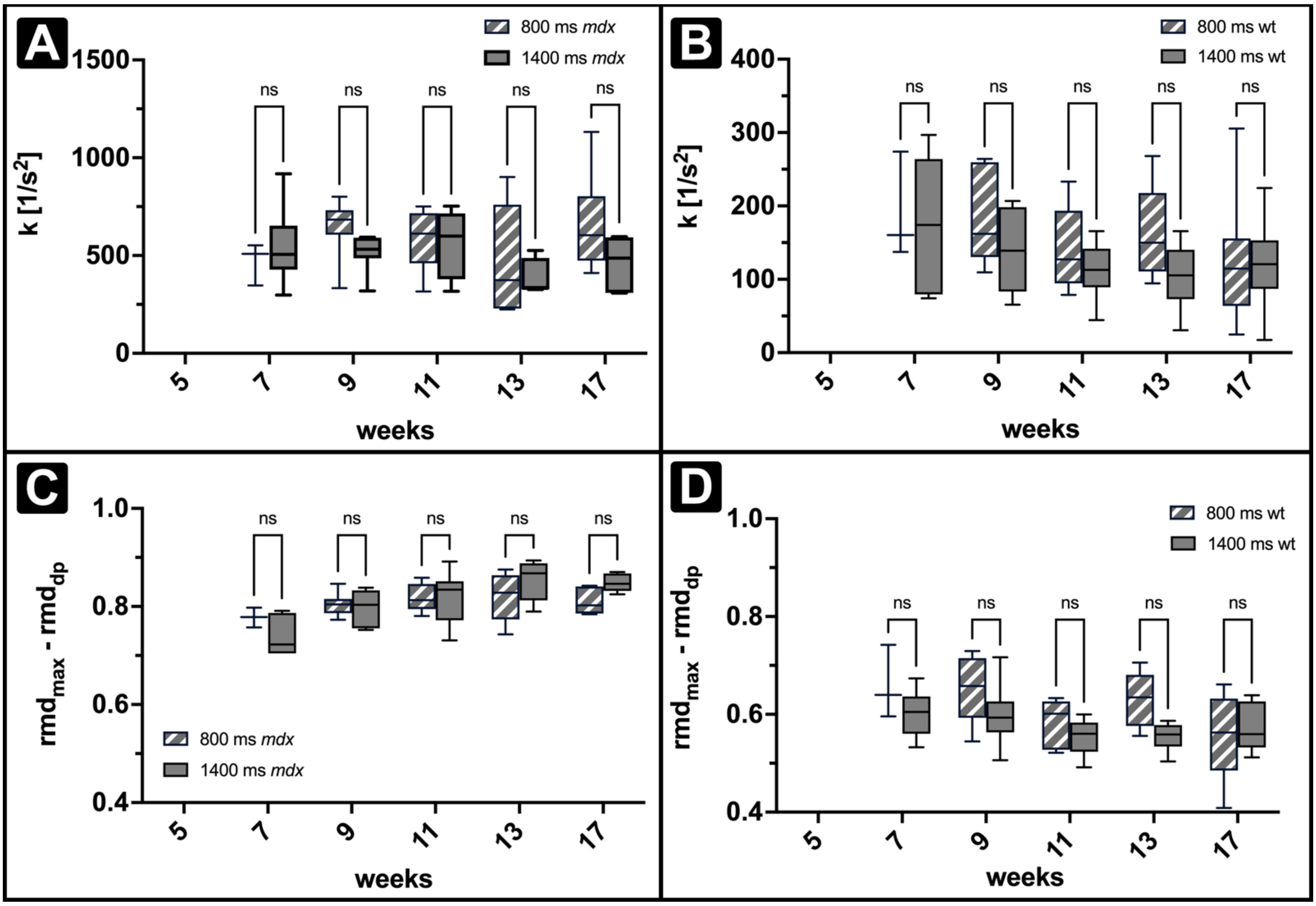

Figure S4. Comparison of expiration constant k (top) and relative fast expiratory phase amplitude (dp) (bottom) at 800 ms and 1400 ms breathing intervals. Expiration constant k is not significantly different between 800 ms and 1400 ms breathing intervals in mdx (**A**) or in wt (**B**) mice. Relative fast expiratory phase amplitude is not significantly different between 800 ms and 1400 ms breathing intervals in mdx (**C**) or in wt (**D**) mice. Box plots show the median with the errors displaying 5-95% intervals. Statistical significance between groups determined by Šídák's multiple comparisons.
